# Respiratory Syncytial Virus Human Experimental Infection Model: Provenance, Production, and Sequence of Low-Passaged Memphis-37 Challenge Virus

**DOI:** 10.1371/journal.pone.0113100

**Published:** 2014-11-21

**Authors:** Young-In Kim, John P. DeVincenzo, Bart G. Jones, Rajeev Rudraraju, Lisa Harrison, Rachel Meyers, Jeff Cehelsky, Rene Alvarez, Julia L. Hurwitz

**Affiliations:** 1 Department of Pediatrics, University of Tennessee Health Science Center, Memphis, Tennessee, United States of America; 2 Department of Microbiology, Immunology and Biochemistry, University of Tennessee Health Science Center, Memphis, Tennessee, United States of America; 3 Children's Foundation Research Institute of Le Bonheur Children's Hospital, Memphis, Tennessee, United States of America; 4 Department of Infectious Diseases, St. Jude Children's Research Hospital, Memphis, Tennessee, United States of America; 5 Alnylam Pharmaceuticals, Cambridge, Massachusetts, United States of America; University of Iowa, United States of America

## Abstract

Respiratory syncytial virus (RSV) is the leading cause of lower respiratory tract infections in children and is responsible for as many as 199,000 childhood deaths annually worldwide. To support the development of viral therapeutics and vaccines for RSV, a human adult experimental infection model has been established. In this report, we describe the provenance and sequence of RSV Memphis-37, the low-passage clinical isolate used for the model's reproducible, safe, experimental infections of healthy, adult volunteers. The predicted amino acid sequences for major proteins of Memphis-37 are compared to nine other RSV A and B amino acid sequences to examine sites of vaccine, therapeutic, and pathophysiologic interest. Human T- cell epitope sequences previously defined by *in vitro* studies were observed to be closely matched between Memphis-37 and the laboratory strain RSV A2. Memphis-37 sequences provide baseline data with which to assess: (i) virus heterogeneity that may be evident following virus infection/transmission, (ii) the efficacy of candidate RSV vaccines and therapeutics in the experimental infection model, and (iii) the potential emergence of escape mutants as a consequence of experimental drug treatments. Memphis-37 is a valuable tool for pre-clinical research, and to expedite the clinical development of vaccines, therapeutic immunomodulatory agents, and other antiviral drug strategies for the protection of vulnerable populations against RSV disease.

## Introduction

Respiratory syncytial virus (RSV) is a paramyxovirus that infects more than 60% of children during the first year of life [1]. This virus is associated with significant morbidity and mortality, particularly among young infants [2]. Globally, RSV infections were estimated to cause 66,000–199,000 deaths in 2005 in children under the age of 5 years, mostly occurring in the developing world, and no vaccine or effective antiviral treatment for RSV disease exists.

Prior to the clinical testing of new vaccines, antivirals, and other novel interventions in infants, safety and efficacy tests should be performed in and proven in consenting adults. However, RSV-directed drug efficacy is difficult to evaluate in healthy adult populations, because natural RSV infections severe enough to prompt a health care concern are relatively rare in adults, producing generally mild symptoms that are difficult to distinguish from those of the common cold. The development of an RSV human adult experimental infection model is therefore imperative to expedite drug paths to licensure and commercialization.

RSV Memphis-37 was isolated from a child with bronchiolitis, characterized, and manufactured for use as a challenge virus in the adult experimental infection model. It supports safe, reproducible, quantifiable, and transient RSV infection and respiratory disease manifestations in adult volunteers. The virus has been used for studies of human RSV disease [3], [4] and the clinical testing of disease inhibitory drugs including anti-inflammatory immunomodulators and passively-transferred antibodies (e.g. MEDI-557 by MedImmune LLC [ClinicalTrials.gov identifier NCT01475305], ALS-008176 by Alios Biopharma, Inc. [ClinicalTrials.gov identifier NCT02094365], ALN-RSV01 by Alnylam Pharmaceuticals [ClinicalTrials.gov identifier NCT00496821], GS-5806 by Gilead Sciences [5], and RV568 by Respivert Ltd. [ClinicalTrials.gov identifier NCT01230645]), as well as pre-clinical research [4], [6]–[10]. Based on results from human adult tests with RSV Memphis-37, antiviral drug products are gaining regulatory approval for testing in high-risk adult populations, infants and children.

In this report, we describe the provenance of Memphis-37. We also compare 11 predicted protein sequences of Memphis-37 to those of other RSV A and B isolates. This information serves as a baseline reference for evaluation of future tests with the experimental RSV infection model. In addition, when escape mutants appear after vaccine or therapeutic drug testing with Memphis-37, results will indicate viral sites that may be targeted when second-generation drugs are developed.

## Results and Discussion

In order to select a challenge virus for a reliable and useful RSV human experimental infection model, children with RSV were first identified from an outpatient urgent care center, emergency department, or from the inpatient area of a large regional pediatric hospital in Memphis, TN. All investigations involving any human subject were approved by the University of Tennessee Health Science Center Internal Review Board. Written informed consent was obtained from parents or legal guardians of the subjects, all of whom were below the age of ascent. From the years 2000–2005, 288 patients without chronic underlying conditions under two years of age who tested positive for RSV by antigen detection (Binax Now and Directigen) were enrolled. Participants were excluded if they had a history of prematurity, cardiac disease, chronic lung disease, immune deficiencies, bacterial co-infections, treatment with corticosteroids, ribavirin, RSV-Ig (Respigam), or palivizumab, or any experimental RSV intervention. The initial nasal aspirates were collected quantitatively as described previously [11] using FDA-approved sterile normal saline for inhalation. The samples were then aliquoted into sterile-sealed polystyrene vials. One fresh aliquot was utilized to quantify RSV by quantitative plaque assay in HEp2 cells without freezing [11], while the remainder of the sample was snap-frozen on dry ice and stored at −80°C. All 288 virus samples were evaluated by RSV strain-specific antibodies, N-gene specific genotype PCR, and quantitative PCR [11], [12].

To manufacture a virus that caused infection and disease similar to natural RSV infection in humans, we kept the viral passage number as low as possible. This minimized nucleic acid mutations caused by RNA-dependent RNA polymerase infidelity and the consequent loss of primary virus features, including patterns of viral protein glycosylation defined by post-translational modifications in human respiratory tract epithelial host cells. Original aliquots of samples from the 288 patients with the six highest viral loads in nasal washes (that were RSV-A defined by both serotyping and genotyping) were taken into a manufacturing suite and processed following Current Good Manufacturing Practices (cGMP). Samples were thawed and plaqued in FDA-approved Vero cell cultures. Each of the six selected viruses produced visible cytopathic effects (CPE), and three individual plaques from each of the six viruses were selected and aliquoted. One aliquot from each of the 18 plaques was placed into a Vero cell serum-free culture to assess quantitative viral growth kinetics. Primary aliquots from cultures exhibiting the most optimal *in vitro* growth kinetics were then manufactured using cGMP guidelines by passage in Vero cell culture roller bottles. Memphis-37 was then selected for final production, and the fill-finish was aliquoted in sterile glass vials and stored at −80°C. The final test product was passaged only five times. Tests for purity and adventitious agents during cGMP production were negative as listed in the FDA guidance documents for live viral vaccine production for human use.

The patient from whom Memphis-37 was originally isolated was a four month old, 5.9 kg, non-breast fed, African-American male who was the 2.6 kg product of a 40 week full term gestation. He was previously healthy and without environmental tobacco smoke exposure. In 2001, he developed respiratory symptoms without fever. On the 5th day of symptoms, the child was hospitalized for bronchiolitis, at which time informed consent and repository consent were obtained, and the patient's first nasal aspirate (from which Memphis-37 strain was isolated) was collected. His mother's prenatal labs were negative for human immunodeficiency virus (HIV) and the surface antigen of the hepatitis B virus (HBsAg), and her standard pre-natal screening labs were unremarkable. She had no history of transfusion or sexually transmitted infection. The patient's initial viral load was >6.78 log plaque forming units (PFU)/ml by fresh quantitative culture on HEp2 cells. The patient's quantitative culture-based viral load declined typically over successive days indicating normal RSV clearance [13]. The patient's viral load by qPCR was initially 7.29 log PFU(e)/ml and declined similarly over successive days. The patient remained hospitalized for four days, did not require supplemental oxygen, never required intensive care or mechanical ventilation, and did not receive any antivirals, passive antibodies or antibiotics.

The Memphis 37 virus was plaqued onto HEp-2 cells to examine morphology. Virus plaquing was performed with the patient's initial nasal aspirate and with the virus that had been passaged five times on Vero cells. The latter sample was also examined by electron microscopy at Advanced Biotechnologies Inc. (Columbia, MD). Plaques were easily countable on both HEp-2 cells and Vero cells. No contaminating adventitious agents including other viruses, yeasts, molds or bacteria were found. Virions were pleomorphic, round or filamentous, and of size consistent with RSV. As expected, small spikes were observed on virion surfaces.

Memphis-37 was amplified once more in HEp2 cells (for a total of six passages) to obtain RNA for Sanger sequencing. The complete sequence was submitted to GenBank (Accession # KM360090). There were several base positions for which major and minor peaks were identified in Sanger electropherograms. Affected positions and major and minor bases in the GenBank sequence were: 4188 (G/A), 4734 (A/G), 6041 (C/T), 8990 (A/C), 10,101 (A/T), 10,102 (A/T), 10,103 (A/T), and 14,102 (G/T). Attention to these heterogeneous sequences may reveal genetic bottlenecks in future infection or transmission studies with the Memphis-37 challenge virus.

The predicted amino acid sequences of RSV Memphis-37 proteins were determined using CLC software (Genomics Workbench, Qiagen) and aligned with nine additional viral sequences from GenBank, including five RSV type A sequences and four RSV type B sequences. Only the major bases described above were used for amino acid predictions. Within the comparator sequences were four laboratory isolates, including RSV A2 and Long, which are routinely used in basic and translational research laboratories [14], [15].

**Figure 1:**
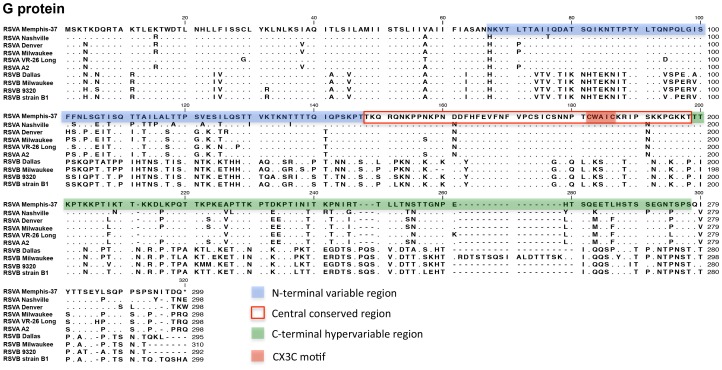
Predicted amino acid sequence for RSV Memphis-37 G protein and alignments. Predicted amino acid sequence is shown for the RSV Memphis-37 G protein. Sequence was aligned with five subtype A viruses (including two laboratory strains) and four subtype B viruses (including two laboratory strains). Virus nomenclature and GenBank Accession numbers used in the alignment are: RSVA Nashville (JX069801.1), RSVA Denver (GU591769.1 [42]), RSVA Milwaukee (JF920069.1 [43]), RSVA VR-26 Long (AY911262.1, Laboratory strain), RSVA A2 (M74568.1, Laboratory strain [15]), RSVB Dallas (JQ582843.1), RSVB Milwaukee (JN032117.1 [43]), RSVB 9320 (AY353550.1, B9320 Laboratory strain), RSV strain B1 (AF013254.1, B1 Laboratory strain [44]). N-terminal variable region: a.a. 67–147, central conserved region: a.a. 148–198, C-terminal hypervariable region: a.a. 199–298, CX3C motif: a.a. 182–186. RSV Memphis-37 sequencing methods: For sequencing purposes, Memphis-37 was taken after five passages and was amplified once more in a T25 flask of HEp-2 cells. Briefly, virus was added to cells in DMEM/0.1% BSA for 1.5 hours. Medium was removed and replaced with EMEM/5% FCS for four days. A lysate was prepared and viral RNA was extracted using a Qiagen viral RNA mini kit. PCR reactions were performed using the TaKaRa One Step RNA PCR Kit (AMV) using 5 µl (145 ng/µl) of viral RNA extracted with Qiagen QIAmp Viral RNA Mini Kit. Forward and reverse oligonucleotides were prepared at concentrations of 100 µM and 1 µl of each pair was used in each reaction. Incubations were at 50°C for 30 min. and 94°C for 2 min. Then 40 cycles were run at 94°C for 30 seconds, 60°C for 30 seconds, and 72°C for 1.5 min. For each sequencing reaction, 40 ng PCR product were mixed with 3.2 pmoles oligonucleotide primer in a final volume of 12 µl and submitted to the Hartwell Center at St. Jude for Sanger sequencing. Sequences were edited and a contig was created in Vector NTI SeqMan. The consensus contig for Memphis-37 was imported into CLC Workbench and predicted amino acid sequences were aligned with other RSV sequences.

**Figure 2:**
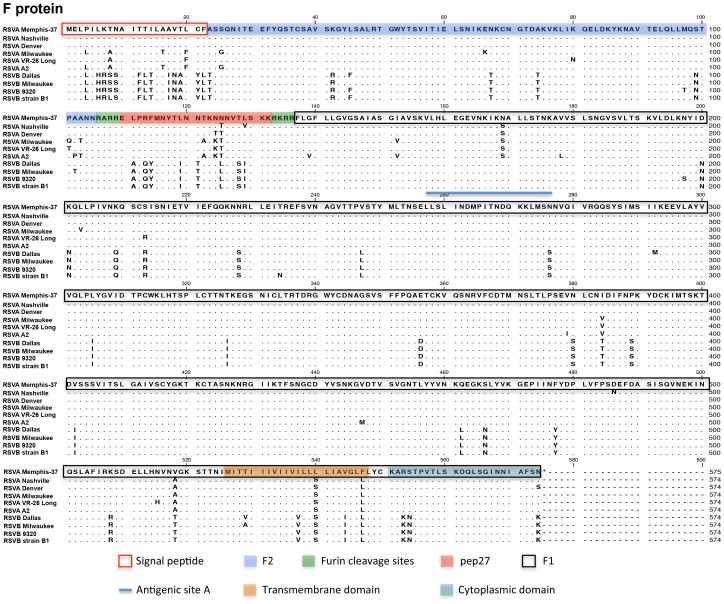
Predicted amino acid sequence for RSV Memphis-37 F protein and alignments. Alignments are as described in Figure 1, but for the F protein. Features of interest are shaded or boxed as indicated below the sequence. The blue line marks an antigenic site A, which is targeted by the monoclonal antibody Palivizumab.

Results are shown for the RSV membrane glycoprotein (G) and fusion (F) protein in Figures 1 and 2, respectively, and for the nine additional proteins in Supplementary Figures (Figure S1. NS-1 protein; Figure S2. NS-2 Protein; Figure S3. N Protein; Figure S4. P protein; Figure S5. M Protein; Figure S6. SH Protein [16]; Figure S7. M2-1 Protein; Figure S8. M2-2 Protein; Figure S9. L protein [15], [17]). Memphis-37 was confirmed to be a type A virus and was subtyped as GA5 based on G protein C-terminal sequences (beginning at a.a. position 210 [18], [19]). GA5 was a common circulating genotype in 2001, the isolation year for Memphis-37 [18], [20].

RSV G (Figure 1) is an attachment protein that adheres virus to its target mammalian cell, in part by binding glycosaminoglycans on the host cell surface [21]. A relatively well-conserved central cysteine loop region (a.a. 148–198) was shared in sequence between Memphis-37 and other RSV type A viruses. This region contains a CX3C motif (CWAIC at a.a. position 182–186) known to modify the host immune response during RSV infection by chemokine mimicry and alteration of leukocyte migration [22]. The CX3C motif binds the fractalkine receptor CX3CR1 and modifies CX3CR1-positive RSV-specific T-cell responses [23]. Antibodies generated toward this central conserved region of RSV G have some ability to cross-neutralize RSV A and B strains [24], [25].

Multiple potential N-linked glycosylation sites (N-X-T or N-X-S when X is not proline) were present in the G protein. The G protein alignment in Figure 1 illustrated the positioning of variable regions known to include sites for neutralizing antibody recognition and associated virus escape [26]. Alignments of predicted amino acid sequences for other RSV proteins (Figure 2 and Figures S1 to S9) showed additional variable regions including those in the C-terminal, extracellular region of SH (including a site of potential N-linked glycosylation, a.a. positions 52-54 in Figure S6), the central region of M2-2, and two regions in L [17].

The RSV F protein (Figure 2) is essential for RSV fusion and entry into its host cell target. The full-length protein F_0_ is cleaved to fragments F_1_ and F_2_ to form the mature F protein. Figure 2 illustrates important features of F including its signal peptide (a.a. 1–22), furin cleavage sites (a.a. 106–109 and 133–136 [27]), pep27 (a.a. 110–136, a small peptide released upon protein cleavage), the transmembrane domain (a.a. 526–548), and the cytoplasmic domain (a.a. 550–574). A key site for virus resistance to a monoclonal antibody that is used as RSV prophylaxis in children (Palivizumab [28]) exists between a.a. positions 257–276 (antigenic site A, indicated by the blue line in Figure 2). Amino acid substitutions in this site that have conferred resistance to Palivizumab in cell culture, animal models, or humans are N262Y/S, N268I, K272E/M/N/Q/T, S275F, and N276Y [29]–[31]. These substitutions were not identified in the Memphis-37 sequence. A more recently described D25 antibody also exhibits potent neutralization of RSV [32]. D25 inhibits F-mediated fusion. This antibody binds a quaternary epitope named antigenic site Ø at the membrane-distal apex of the pre-fusion F trimer. It interacts with the α4 helix (F1 residues 196–210) and forms intermolecular hydrogen bonds with F2 residues 63, 65, 66, and 68. Antibody contact points are shared between RSV Memphis 37 and A2, but not between RSV A and B isolates. Continued research studies with neutralizing antibodies and Memphis 37 are encouraged to unravel mechanisms of virus inhibition, while defining new strategies for RSV control.

RSV-specific CD4-positive and CD8-positive T lymphocytes partner with antibodies to inhibit virus infections by producing cytokines, killing virus infected targets, and regulating innate and adaptive effector functions. We examined human T-cell epitope sequences that had previously been described by *in vitro* studies, mapped onto the RSV A2 sequence [33]–[41]. Table 1 shows that these epitope sequences were almost entirely matched between Memphis-37 and RSV A2. All epitopes within the G, N, M, M2, and NS proteins showed 100% sequence homology between the two strains. A T-cell epitope sequence flanking the CXC3 motif in the RSV G protein was perfectly matched between RSV A2 and Memphis-37 (a.a. 162–175). Only a few epitope sequences within the F proteins, which are bolded in Table 1, were observed to be different. As studies with the experimental infection model progress, attention should be paid to variable sites and regions, which may mark positions of virus escape from candidate vaccines and therapeutic drugs.

**Table 1:** Comparison of human CD4 and CD8 T-lymphocyte epitope sequences between respiratory syncytial virus Memphis-37 and A2 strains.

	RSV protein	Amino acid position	Peptide sequence*			% Homology between peptides	
			RSV-Memphis-37		RSV-A2		
**CD4**	G	162−175		DFHFEVFNFVPCSI	DFHFEVFNFVPCSI		100
	F	7−30		K**T**NAITTIL**A**AVT**L**CFAS**S**QNITE	KANAITTILTAVTFCFASGQNITE		83
	F	25−42		**S**QNITEEFYQSTCSAVSK	GQNITEEFYQSTCSAVSK		94
	F	43−60		GYLSALRTGWYTSVITIE	GYLSALRTGWYTSVITIE		100
	F	49−72		RTGWYTSVITIELSNIKENKCNGT	RTGWYTSVITIELSNIKENKCNGT		100
	F	55−72		SVITIELSNIKENKCNGT	SVITIELSNIKENKCNGT		100
	F	73−90		DAKVKLIKQELDKYKNAV	DAKVKLIKQELDKYKNAV		100
	F	85−102		KYKNAVTELQLLMQSTP**A**	KYKNAVTELQLLMQSTPP		94
	F	109−132		RELPRFMNYTLNN**T**K**NN**NVTLSKK	RELPRFMNYTLNNAKKTNVTLSKK		88
	F	175−192		NKAVVSLSNGVSVLTSKV	NKAVVSLSNGVSVLTSKV		100
	F	193−210		LDLKNYIDKQLLPIVNKQ	LDLKNYIDKQLLPIVNKQ		100
	F	229−252		RLLEITREFSVNAGVTTPVSTYML	RLLEITREFSVNAGVTTPVSTYML		100
	F	265−288		PITNDQKKLMSNNVQIVRQQSYSI	PITNDQKKLMSNNVQIVRQQSYSI		100
	F	295−318		EVLAYVVQLPLYGVIDTPCWKLHT	EVLAYVVQLPLYGVIDTPCWKLHT		100
	F	337−360		TDRGWYCDNAGSVSFFPQAETCKV	TDRGWYCDNAGSVSFFPQAETCKV		100
	F	391−408		YDCKIMTSKTDVSSSVIT	YDCKIMTSKTDVSSSVIT		100
	F	409−426		SLGAIVSCYGKTKCTASN	SLGAIVSCYGKTKCTASN		100
	F	427−444		KNRGIIKTFSNGCDYVSN	KNRGIIKTFSNGCDYVSN		100
	F	457−486		YYVNKQEGKSLYVKGEPIINFYDPLVFPSD	YYVNKQEGKSLYVKGEPIINFYDPLVFPSD		100
	F	493−516		SQVNEKINQSLAFIRKSDELLHNV	SQVNEKINQSLAFIRKSDELLHNV		100
	F	517−534		N**V**GKSTTNIMITTIIIVI	NAGKSTTNIMITTIIIVI		94
	F	541−558		LIAVGL**F**LYCKARSTPVT	LIAVGLLLYCKARSTPVT		94
**CD8**	F	8−17		**T**NAITTIL**A**A	ANAITTILTA		80
	F	93−102		LQLLMQSTPA	LQLLMQSTPA		100
	F	106–113		RARRELPRF	RARRELPRF		100
	F	109–118		RELPRFMNYT	RELPRFMNYT		100
	F	260–269		LINDMPITND	LINDMPITND		100
	F	273–282		LMSNNVQIVR	LMSNNVQIVR		100
	F	285–294		SYSIMSIIKE	SYSIMSIIKE		100
	F	374–383		TLPSEVNLCN	TLPSEVNLCN		100
	F	388–397		NPKYDCKIMT	NPKYDCKIMT		100
	F	519–528		GKST**T**NIMIT	GKSTINIMIT		90
	F	521–530		ST**T**NIMITTI	STINIMITTI		90
	F	542–550		IAVGL**F**LYC	IAVGLLLYC		89
	N	46–59		KLCGMLLITEDANH	KLCGMLLITEDANH		100
	N	232–245		STRGGSRVEGIFAG	STRGGSRVEGIFAG		100
	N	250–263		AYGAGQVMLRWGVL	AYGAGQVMLRWGVL		100
	N	253–266		AGQVMLRWGVLAKS	AGQVMLRWGVLAKS		100
	N	256–269		VMLRWGVLAKSVKN	VMLRWGVLAKSVKN		100
	N	298–311		AGFYHILNNPKASL	AGFYHILNNPKASL		100
	N	306–314		NPKASLLSL	NPKASLLSL		100
	M	195–203		IPYSGLLLV	IPYSGLLLV		100
	M	229–237		YLEKESIYY	YLEKESIYY		100
	M2-1	64–72		AELDRTEEY	AELDRTEEY		100
	M2-1	151–159		RLPADVLKK	RLPADVLKK		100
	NS1	41–49		LAKAVIHTI	LAKAVIHTI		100

Legend: *Differences between Memphis-37 and RSV A2 are bolded.

In conclusion, we have described the provenance, production, and sequences of Memphis-37. These data serve as valuable reference material for the evaluation of RSV vaccines and therapeutics in the adult human experimental infection model. Memphis 37 has already supported RSV research *in vitro* and *in vivo*, including studies in lambs, monkeys and man Clinical studies test numerous drug products, including candidates for RSV prophylaxes in children (e.g. monoclonal antibodies) and RSV treatments (e.g. small molecules and siRNAs). Future research will continue to examine the characteristics of Memphis 37 in challenge, transmission and serological studies while expediting the development of clinical vaccines and therapeutic drug strategies for the protection of vulnerable populations against RSV disease.

## Supporting Information

Figure S1 **Predicted amino acid sequence for RSV Memphis-37 NS1 protein and alignments.** Alignments are as described for Figure 1, but for the NS1 protein.
(TIF)

Figure S2 **Predicted amino acid sequence for RSV Memphis-37 NS2 protein and alignments.** Alignments are as described for Figure 1, but for the NS2 protein.
(TIF)

Figure S3 **Predicted amino acid sequence for RSV Memphis-37 N protein and alignments.** Alignments are as described for Figure 1, but for the N protein.
(TIF)

Figure S4 **Predicted amino acid sequence for RSV Memphis-37 P protein and alignments.** Alignments are as described for Figure 1, but for the P protein.
(TIF)

Figure S5 **Predicted amino acid sequence for RSV Memphis-37 M protein and alignments.** Alignments are as described for Figure 1, but for the M protein.
(TIF)

Figure S6 **Predicted amino acid sequence for RSV Memphis-37 SH protein and alignments.** Alignments are as described for Figure 1, but for the SH protein. Transmembrane domain: a.a. 23–41, Potential site for glycosylation: a.a. 52–54.
(TIF)

Figure S7 **Predicted amino acid sequence for RSV Memphis-37 M2-1 protein and alignments.** Alignments are as described for Figure 1, but for the M2-1 protein.
(TIF)

Figure S8 **Predicted amino acid sequence for RSV Memphis-37 M2-2 protein and alignments.** Alignments are as described for Figure 1, but for the M2-2 protein.
(TIF)

Figure S9 **Predicted amino acid sequence for RSV Memphis-37 L protein and alignments.** Alignments are as described for Figure 1, but for the L protein.
(PDF)
