# Supplementary figures and images for: Respiratory Syncytial Virus Human Experimental Infection Model: Provenance, Production, and Sequence of Low-Passaged Memphis-37 Challenge Virus
Source: PLoS One. 2014 Nov 21;9(11):e113100. doi: 10.1371/journal.pone.0113100 (PMC4240712; doi:10.1371/journal.pone.0113100)

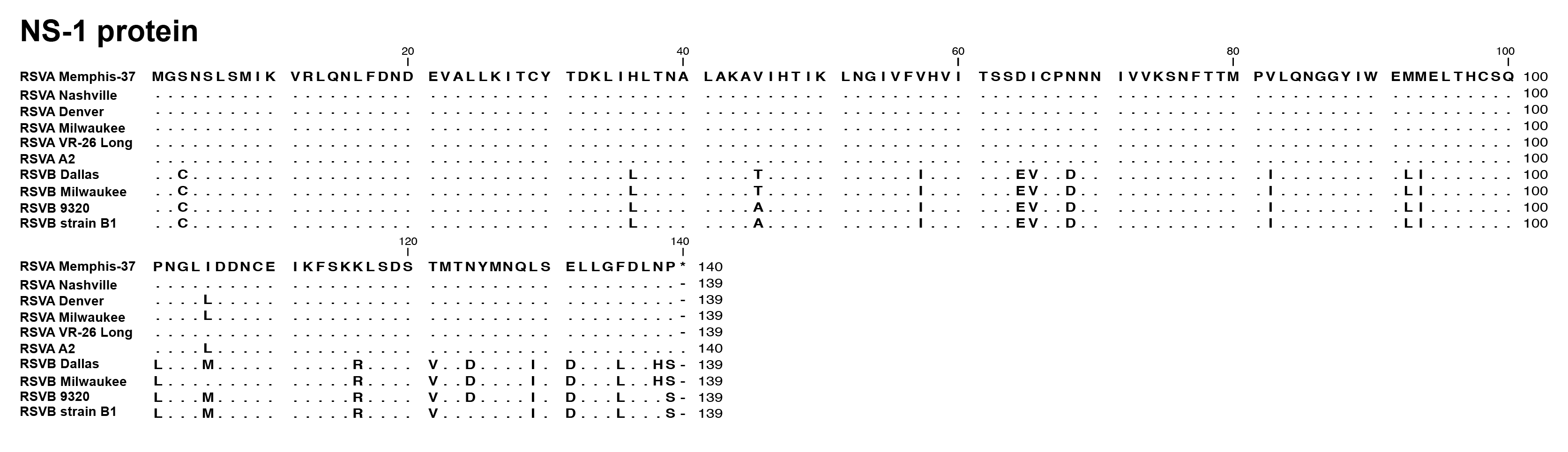

Supplement: Figure S1 — Predicted amino acid sequence for RSV Memphis-37 NS1 protein and alignments. Alignments are as described for Figure 1, but for the NS1 protein. (TIF) [file pone.0113100.s001.tif]

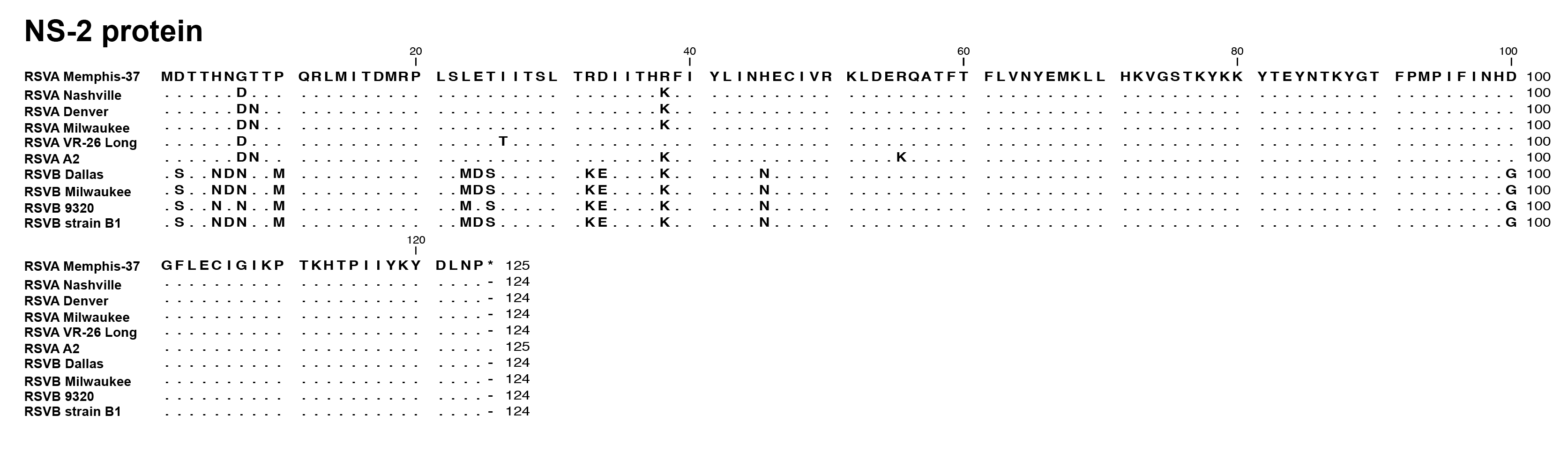

Supplement: Figure S2 — Predicted amino acid sequence for RSV Memphis-37 NS2 protein and alignments. Alignments are as described for Figure 1, but for the NS2 protein. (TIF) [file pone.0113100.s002.tif]

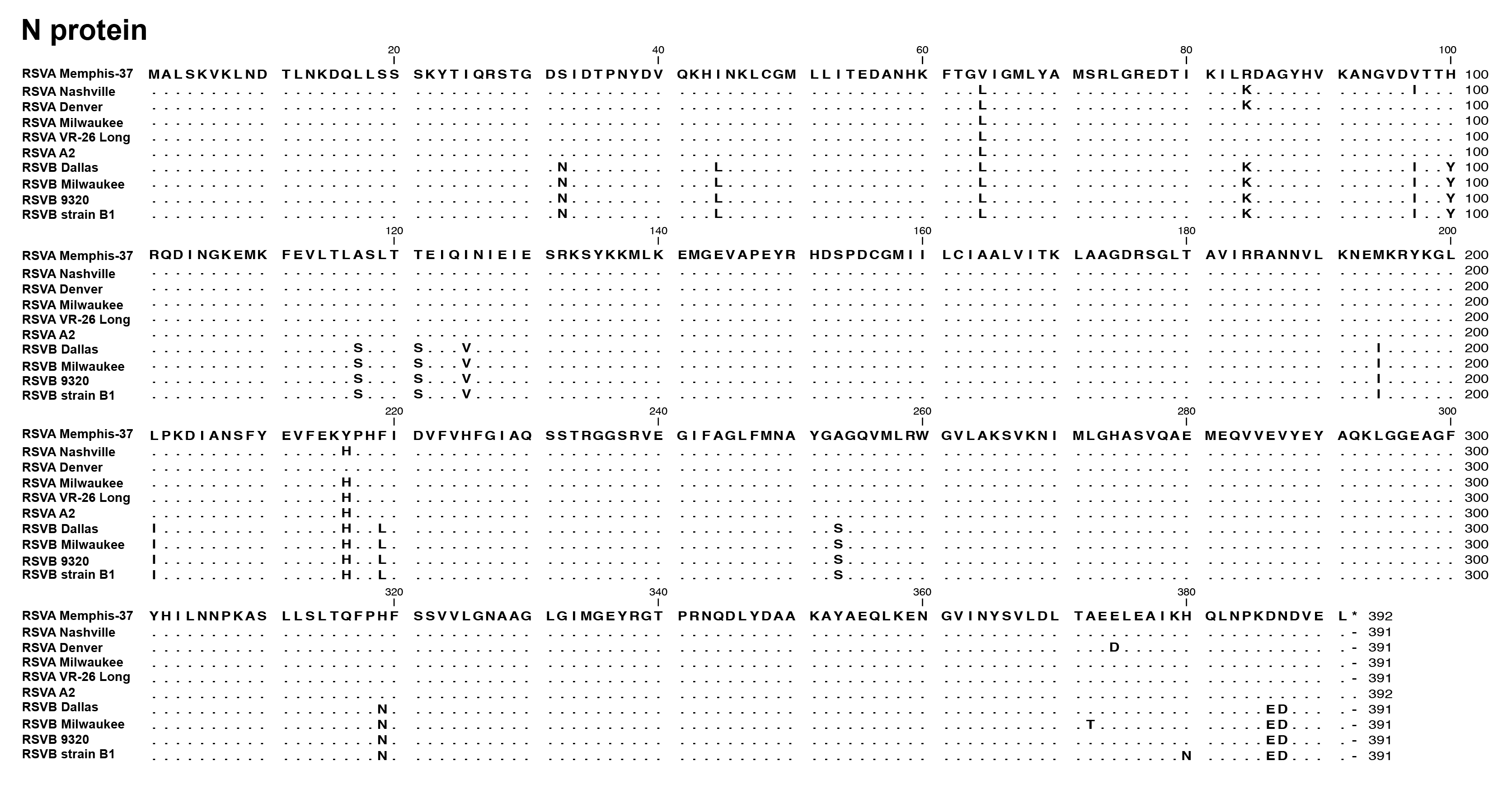

Supplement: Figure S3 — Predicted amino acid sequence for RSV Memphis-37 N protein and alignments. Alignments are as described for Figure 1, but for the N protein. (TIF) [file pone.0113100.s003.tif]

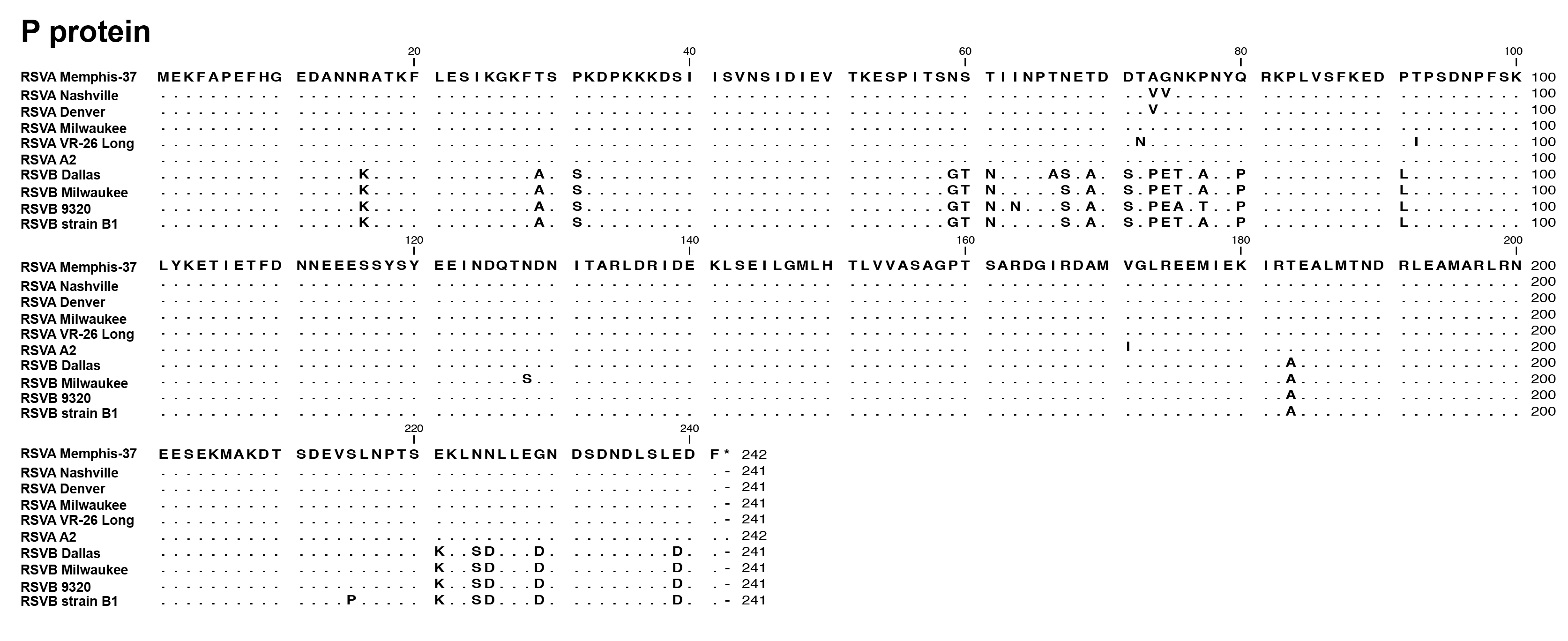

Supplement: Figure S4 — Predicted amino acid sequence for RSV Memphis-37 P protein and alignments. Alignments are as described for Figure 1, but for the P protein. (TIF) [file pone.0113100.s004.tif]

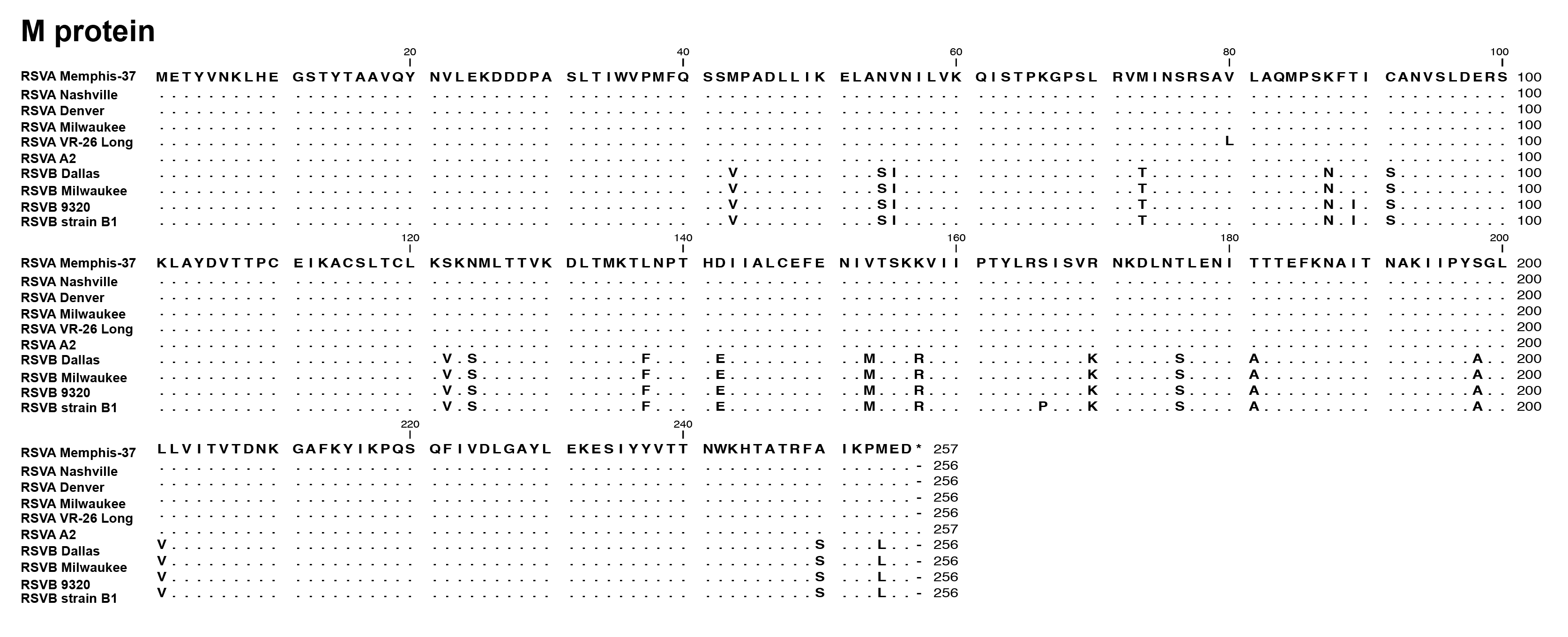

Supplement: Figure S5 — Predicted amino acid sequence for RSV Memphis-37 M protein and alignments. Alignments are as described for Figure 1, but for the M protein. (TIF) [file pone.0113100.s005.tif]

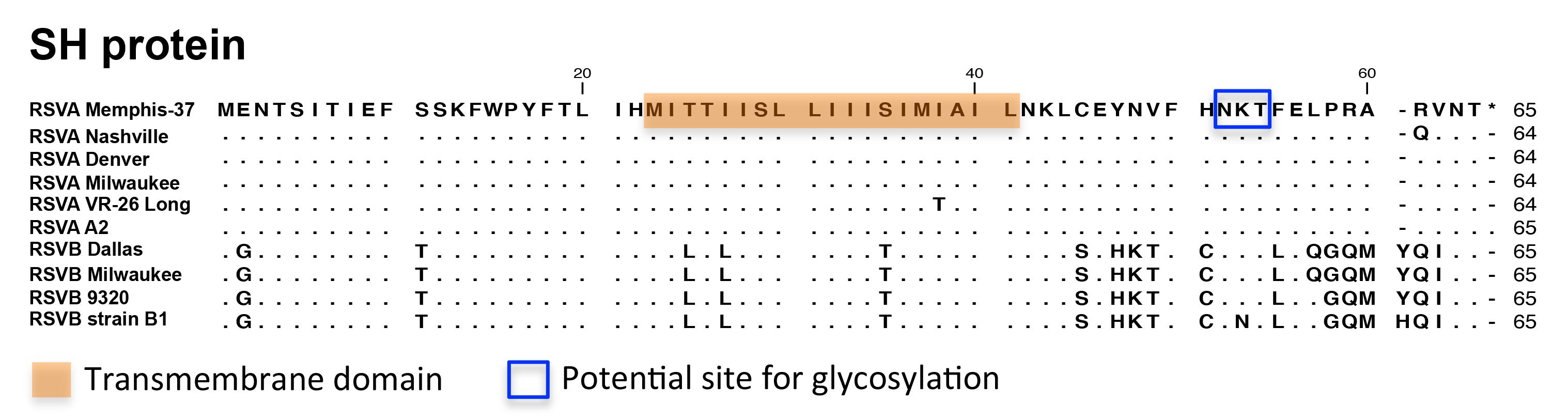

Supplement: Figure S6 — Predicted amino acid sequence for RSV Memphis-37 SH protein and alignments. Alignments are as described for Figure 1, but for the SH protein. Transmembrane domain: a.a. 23–41, Potential site for glycosylation: a.a. 52–54. (TIF) [file pone.0113100.s006.tif]

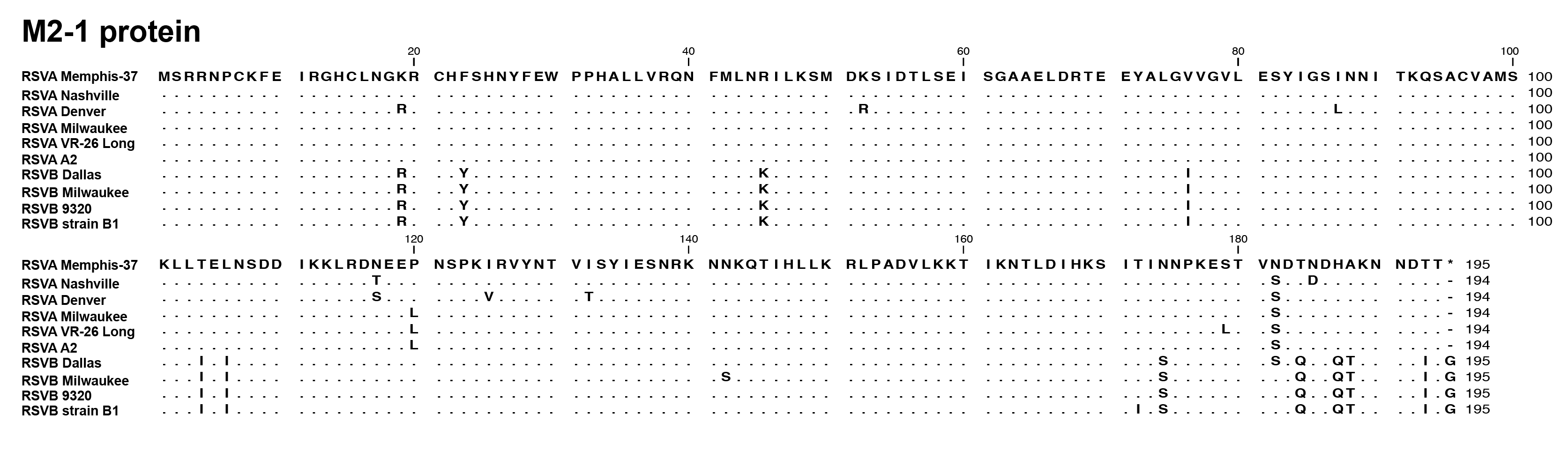

Supplement: Figure S7 — Predicted amino acid sequence for RSV Memphis-37 M2-1 protein and alignments. Alignments are as described for Figure 1, but for the M2-1 protein. (TIF) [file pone.0113100.s007.tif]

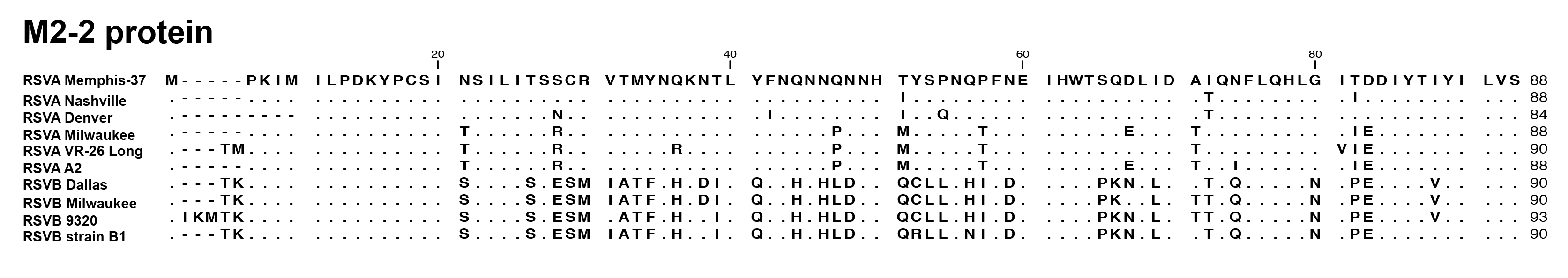

Supplement: Figure S8 — Predicted amino acid sequence for RSV Memphis-37 M2-2 protein and alignments. Alignments are as described for Figure 1, but for the M2-2 protein. (TIF) [file pone.0113100.s008.tif]
