# Supplementary material for: Respiratory Syncytial Virus Human Experimental Infection Model: Provenance, Production, and Sequence of Low-Passaged Memphis-37 Challenge Virus
Source: PLoS One. 2014 Nov 21;9(11):e113100. doi: 10.1371/journal.pone.0113100 (PMC4240712; doi:10.1371/journal.pone.0113100)
Supplement: Figure S9 — Predicted amino acid sequence for RSV Memphis-37 L protein and alignments. Alignments are as described for Figure 1, but for the L protein. (PDF) [file pone.0113100.s009.pdf]

L protein

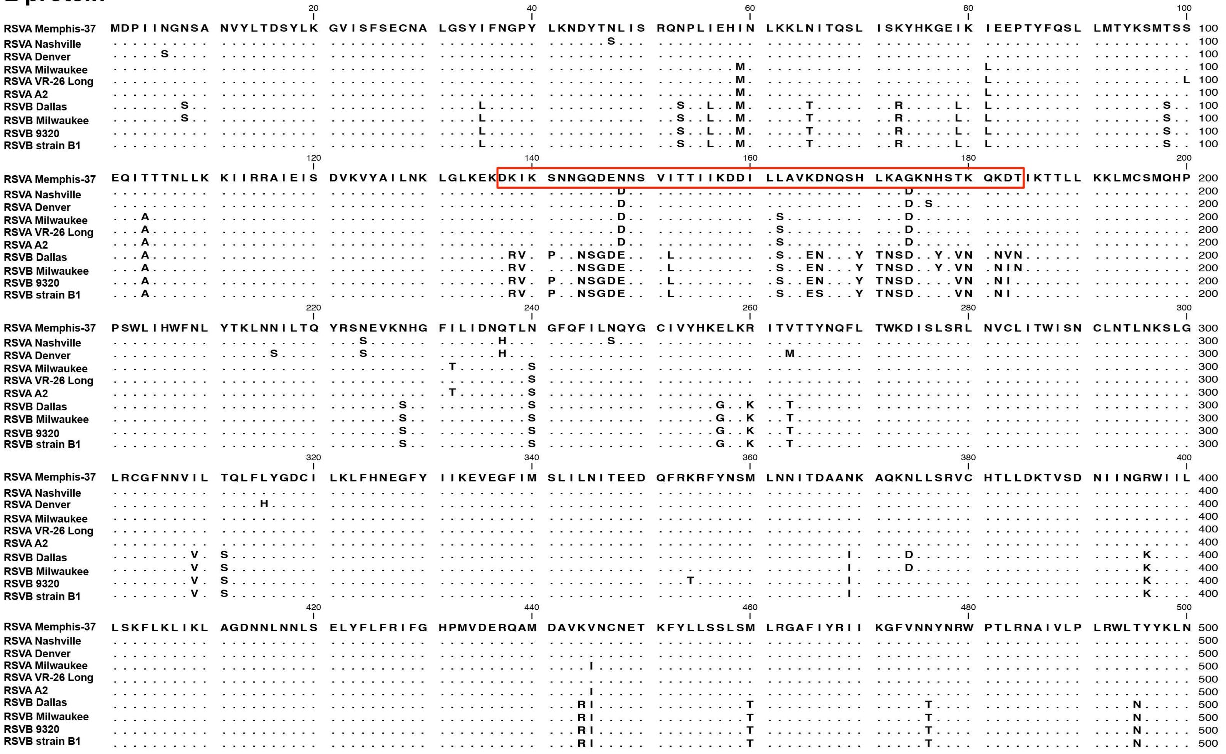

Variable region

L protein

```
                                               520        540        560        580        600
RSVA Memphis-37   TYPSLLELTE RDLIVLSGLR FYREFRLPKK VDLEMIINDK AISPPKNLIW TSFPRNYMPS HIQNYIEHEK LKFSESDKSR RVLEYYLRDN KFNECDLYNC 600
RSVA Nashville    .......... .......... .......... .......... .......... .......... .......... .......... .......... .......... 600
RSVA Denver       .......... .......... .......... .......... .......... .......... .......... .......... .......... .......... 600
RSVA Milwaukee    .......... .......... .......... .......... .......... .......... .......... .......... .......... .......... 600
RSVA VR-26 Long   .......... .......... .......... .......... .......... .......... .......... .......... .......... .......... 600
RSVA A2           .......... .......... .......... .......... .......... .......... .......... .......... .......... .......... 600
RSVB Dallas       .......I.. N...I..... .....H.... .......... ......D... .......... .......... .......R.. .......... .......... 600
RSVB Milwaukee    .......I.. N...I..... .....H.... .......... ......D... .......... .......... .......R.. .......... .......... 600
RSVB 9320         .......I.. N...I..... .....H.... .......... ......D... .......... .......... .......R.. .......... .......... 600
RSVB strain B1    .......I.. N...I..... .....H.... .......... ......D... .......... .......... .......R.. .......... .......... 600

                                               620        640        660        680        700
RSVA Memphis-37   VVNQSYLNNP NHVVSLTGKE RELSVGRMFA MQPGMFRQVQ ILAEKMIAEN ILQFFPESLT RYGDLELQKI LELKAGISNK SNRYNDNYNN YISKCSIITD 700
RSVA Nashville    .......... .......... .......... .......... .......... .......... .......... .......... .......... .......... 700
RSVA Denver       .......... .......... .......... .......... .......... .......... .......... .......... .......... .......... 700
RSVA Milwaukee    .......... .......... .......... .......... .......... .......... .......... .......... .......... .......... 700
RSVA VR-26 Long   .......... .......... .......... .......... .......... .......... .......... .......... .......... .......... 700
RSVA A2           .......... .......... .......... .......... .......... .......... .......... .......... .......... .......... 700
RSVB Dallas       .........S .......... .......... ........I. .......... .......... .......... .......... .......... .......... 700
RSVB Milwaukee    .........S .......... .......... ........I. .......... .......... .......... .......... .......... .......... 700
RSVB 9320         .........S .......... .......... ........I. .......... .......... .......... .......... .......... .......... 700
RSVB strain B1    .........S .......... .......... ........I. .......... .......... .......... .......... .......... .......... 700

                                               720        740        760        780        800
RSVA Memphis-37   LSKFNQAFRY ETSCICSDVL DELHGVQSLF SWLHLTIPHV TIICTYRHAP PYIRDHIVDL NNVDEQSGLY RYHMGGIEGW CQKLWTIEAI SLLDLISLKG 800
RSVA Nashville    .......... .......... .......... .......... .......... .......... .......... .......... .......... .......... 800
RSVA Denver       .......... .......... .......... .......... .......... ...K.P.... .......... .......... .......... .......... 800
RSVA Milwaukee    .......... .......... .......... .......... .......... ...G...... .......... .......... .......... .......... 800
RSVA VR-26 Long   .......... .......... .......... .......... .......... .......... .......... .......... .......... .......... 800
RSVA A2           .......... .......... .......... .......... .......... ...G...... .......... .......... .......... .......... 800
RSVB Dallas       .......... .......... .......... ........L. .......... .F.K..V.N. .E........ .......... .......... .......... 800
RSVB Milwaukee    ........X. .......... .......... ........L. .......... .F.K..V.N. .E........ .......... .......... .......... 800
RSVB 9320         .......... .......... .......... ........L. .......... .F.K..V.N. .E........ .......... .......... .......... 800
RSVB strain B1    .......... .......... .......... ........L. .......... .F.K..V.N. .E........ .......... .......... .......... 800

                                               820        840        860        880        900
RSVA Memphis-37   KFSITALING DNQSIDISKP VRLMEGQTHA QADYLLALNS LKLLYKEYAG IGHKLKGTET YISRDMQFMS KTIQHNGVYY PASIKKVLRV GPWINTILDD 900
RSVA Nashville    .......... .......... .......... .......... .......... .......... .......... .......... .......... .......... 900
RSVA Denver       .......... .......... .......... .......... .......... .......... .......... .......... .......... .......... 900
RSVA Milwaukee    .......... .......... I......... .......... .......... .......... .......... .......... .......... .......... 900
RSVA VR-26 Long   .......... .......... .......... .......... .......... .......... .......... .......... .......... .......... 900
RSVA A2           .......... .......... I......... .......... .......... .......... .......... .......... .......... .......... 900
RSVB Dallas       .......... .......... ...I...... .......... .......... .......... .......... .......... .......... .......... 900
RSVB Milwaukee    .......... .......... ...I...... .......... .......... .......... .......... .......... .......... .......... 900
RSVB 9320         .......... .......... ...I...... .......... .......... .......... .......... .......... .......... .......... 900
RSVB strain B1    .......... .......... ...I...... .......... .......... .......... .......... .......... .......... .......... 900

                                               920        940        960        980      1,000
RSVA Memphis-37   FKVSLESIGS LTQELEYRGE SLLCSLIFRN VWLYNQIALQ LKNHALCNNK LYLDILKVLK HLKTFFNLDN IDTALTLYMN LPMLFGGGDP NLLYRSFYRR 1000
RSVA Nashville    .......... .......... .......... .......... .......... .......... ...S...... .......... .......... .......... 1000
RSVA Denver       .......... .......... .......... .......... .......... .......... .......... .......... .......... .......... 1000
RSVA Milwaukee    .......... .......... .......... .......... .......... .......... .......... .......... .......... .......... 1000
RSVA VR-26 Long   .......... .......... .......... .......... .......... .......... .......... .......... .......... .......... 1000
RSVA A2           .......... .......... .......... .......... .......... .......... .......... .......... .......... .......... 1000
RSVB Dallas       .......... .......... .......... I......... .R........ .......... .........S .....S.... .......... .......... 1000
RSVB Milwaukee    .......... .......... .......... I......... .R........ .......... .........S .....S.... .......... .......... 1000
RSVB 9320         .......... .......... .......... I......... .R.....H.. .......... .........S ..M..S.... .......... .......... 1000
RSVB strain B1    .......... .......... .......... I......... .R........ .......... .........S ..M..S.... .......... .......... 1000
```

L protein

```
                                          1,020                 1,040                 1,060                 1,080                 1,100
RSVA Memphis-37    TPDFLTEAIV HSVFILSYYT NHDLKDKLQD LSDDRLNKFL TCIITFDKNP NAEFVTLMRD PQALGSERQA KITSEINRLA VTEVLSTAPN KIFSKSAQHY 1100
RSVA Nashville     .......... ....V..... .......... .......... .......... .......... .......... .......... ...I...... .......... 1100
RSVA Denver        .......... .......... .......... .......... .......... .......... .......... .......... .......... .......... 1100
RSVA Milwaukee     .......... .......... .......... .......... .......... .......... .......... .......... .......... .......... 1100
RSVA VR-26 Long    .......... .......... .......... .......... .......... .......... .......... .......... .......... .......... 1100
RSVA A2            .......... .......... .......... .......... .......... .......... .......... .......... .......... .......... 1100
RSVB Dallas        .......... ....V..... G...Q..... .P........ ..V....... .......... .......... .......... ......I... .......... 1100
RSVB Milwaukee     .......... ....V..... G...X..... .P........ ..V....... .......... .......... .......... ......I... .......... 1100
RSVB 9320          .......... ....V..... G...Q..... .P........ .......... .......... .......... .......... ......I... .......... 1100
RSVB strain B1     .......... ....V..... G...Q..... .P........ ..V....... .......... .......... .......... ......I... .......... 1100

                                          1,120                 1,140                 1,160                 1,180                 1,200
RSVA Memphis-37    TTTEIDLNDI MQNIEPTYPH GLRVVYESLP FYKAEKIVNL ISGTKSITNI LEKTSAIDLT DIDRATEMMR KNITLLIRIF PLDCNRDKRE ILSMENLSIT 1200
RSVA Nashville     .......... .......... .......... .......... .......... .......... .....V.... .......... .......... .......... 1200
RSVA Denver        .......... .......... .......... .......... .......... .......... .......... ........LL .......... .......... 1200
RSVA Milwaukee     .......... .......... .......... .......... .......... .......... .......... .........L .......... .......... 1200
RSVA VR-26 Long    .......... .......... .......... .......... .......... .......... .......... .........L .......... .......... 1200
RSVA A2            .......... .......... .......... .......... .......... .......... .......... .........L .......... .......... 1200
RSVB Dallas        .......... .......... .......... .......... .......... ........T. ..N...D... .........L .....K.... L..L...... 1200
RSVB Milwaukee     .......... .......... .......... .......... .......... ........T. ..N...D... .........L .....K.... L..L...... 1200
RSVB 9320          .......... .......... .......... .......... .......... ........T. ..N...D... .........L .....K.... L..L...... 1200
RSVB strain B1     .......... .......... .......... .......... .......... ........T. ..N...D... .........L .....K.... L..L...... 1200

                                          1,220                 1,240                 1,260                 1,280                 1,300
RSVA Memphis-37    ELSKYVRERS WSLSNIVGVT SPSIMYTMDI KYTTSTIASG IIIEKYNVNS LTRGERGPTK PWVGSSTQEK KTMPVYNRQV LTKKQRDQID LLAKLDWVYA 1300
RSVA Nashville     .......... .......... .......... .......... .......... .......... .......... .......... .......... .......... 1300
RSVA Denver        .......... .......... .......... .......... .......... .......... .......... .......... .......... .......... 1300
RSVA Milwaukee     .......... .......... .......... .......S.. .......... .......... .......... .......... .......... .......... 1300
RSVA VR-26 Long    .......... .......... .......... .......... .......... .......... .......... .......... .......... .......... 1300
RSVA A2            .......... .......... .......... .......S.. .......... .......... .......... .......... .......... .......... 1300
RSVB Dallas        .......... .......... .....F.... .......... .........G .......... .......... .......... .......... .......... 1300
RSVB Milwaukee     .......... .......... .....F.... .......... .........G .......... .......... .......... .......... .......... 1300
RSVB 9320          .......... .......... .....F.... .......... .......... .......... .......... .......... .......... .......... 1300
RSVB strain B1     .......... .......... .....F.... .......... .......... .......... .......... .......... .......... .......... 1300

                                          1,320                 1,340                 1,360                 1,380                 1,400
RSVA Memphis-37    SIDNKDEFME ELSIGTLGLT YEKAKKLFPQ YLSVNYLHRL TVSSRPCEFP ASIPAYRTTN YHFDTSPINR ILTEKYGDED IDIVFQNCIS FGLSLMSVVE 1400
RSVA Nashville     .......... .......... .......... .......... .......... .......... .......... .......... .......... .......... 1400
RSVA Denver        .......... .......... .......... .......... .......... .......... .......... .......... .......... .......... 1400
RSVA Milwaukee     .......... .......... .......... .......... .......... .......... .......... .......... .......... .......... 1400
RSVA VR-26 Long    .......... .......... .......... .......... .......... .......... .......... .......... .......... .......... 1400
RSVA A2            .......... .......... .......... .......... .......... .......... .......... .......... .......... .......... 1400
RSVB Dallas        .V........ ...T.....S .......... .......... .......... .......... .........H V......... .......... .......... 1400
RSVB Milwaukee     .......... ...T.....S .......... .......... .......... .......... .........H L......... .......... .......... 1400
RSVB 9320          .......... ...T.....S .......... .......... .......... .......... .........H V......... .......... .......... 1400
RSVB strain B1     .......... ...T.....S .......... .......... .......... .......... .........H V......... .......... .......... 1400

                                          1,420                 1,440                 1,460                 1,480                 1,500
RSVA Memphis-37    QFTNVCPNRI ILIPKLNEIH LMKPPIFTGD VDIHKLKQVI QKQHMFLPDK ISLTQYVELF LSNKTLKSGS HVNSNLILAH KISDYFHNTY ILSTNLAGHW 1500
RSVA Nashville     .......... .......... .......... .......... .......... .......... .......... .......... .......... .......... 1500
RSVA Denver        ....A..... .......... .......... .......... .......... .......... .......... N......... .......... .......... 1500
RSVA Milwaukee     .......... .......... .......... .......... .......... .......... .......... .......... .......... .......... 1500
RSVA VR-26 Long    .......... .......... .......... .......... .......... .......... .......... .......... .......... .......... 1500
RSVA A2            .......... .......... .......... .......... .......... .......... .......... .......... .......... .......... 1500
RSVB Dallas        ....I..... .......... .......... ...I...... .......... .......... ....A..... .I......V. .M......A. .......... 1500
RSVB Milwaukee     ....I..... .......... .......... ...I...... .......... .......... ....A..... .I......V. .M......A. .......... 1500
RSVB 9320          ....I..... .......... .......... ...I...... .......... .......... ....A..... .I......V. .M......D. .......... 1500
RSVB strain B1     ....I..... .......... .......... ...I...... .......... .......... ....A..... NI......V. .M......A. .......... 1500
```

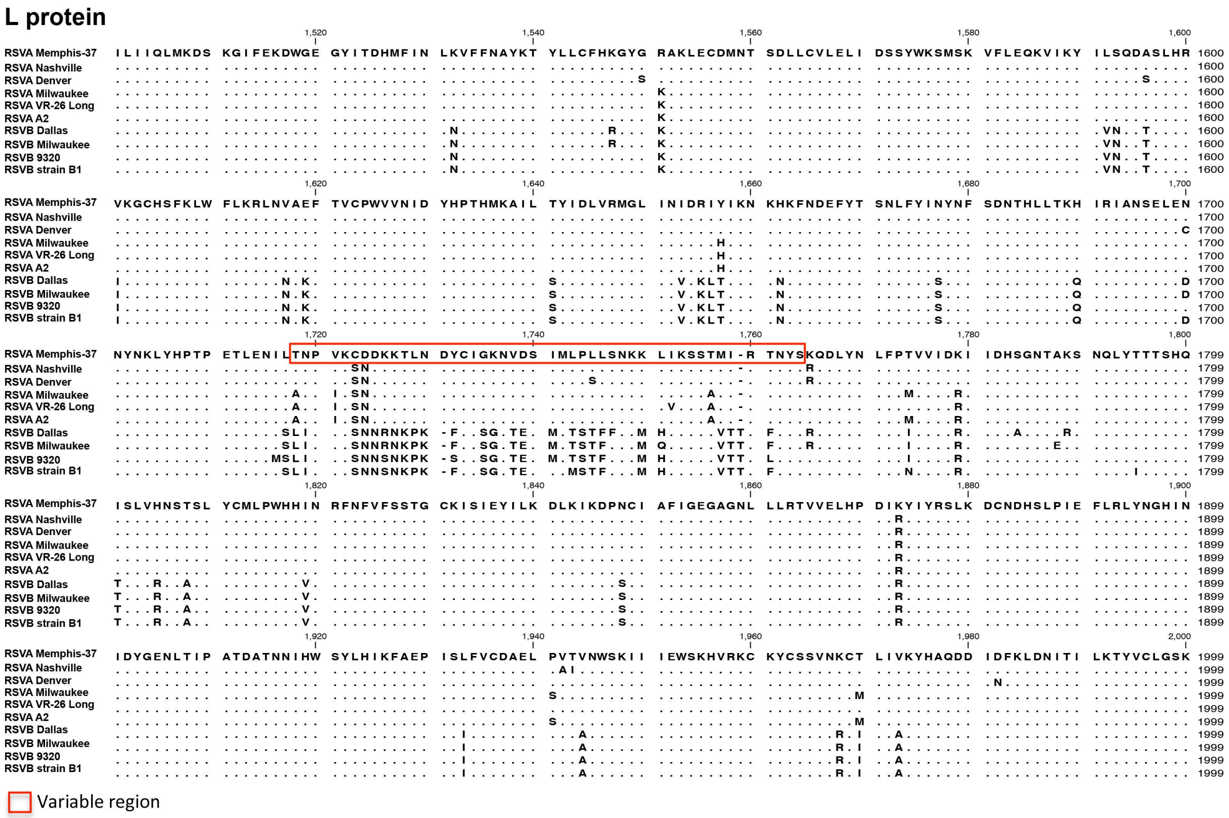
L protein
RSVA Memphis-37
RSVA Nashville
RSVA Denver
RSVA Milwaukee
RSVA VR-26 Long
RSVA A2
RSVB Dallas
RSVB Milwaukee
RSVB 9320
RSVB strain B1
Variable region

L protein

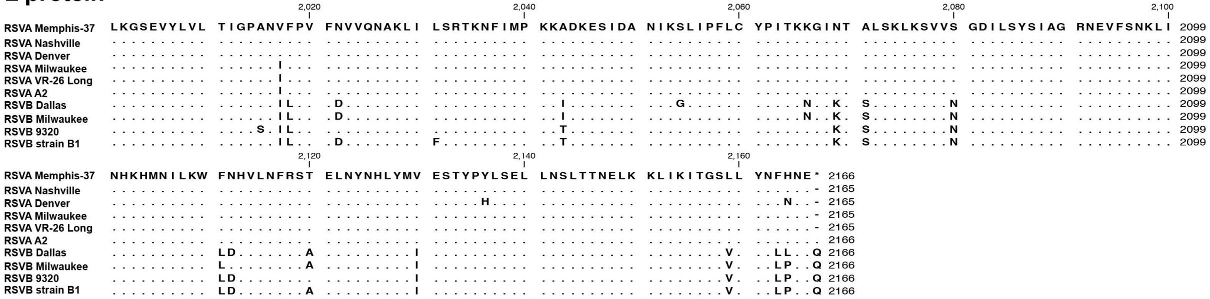

2,020
2,040
2,060
2,080
2,100
RSVA Memphis-37
LKGSEVYLVL TIGPANVFPV FNVVQNAKLI LSRTKNFIMP KKADKESIDA NIKSLIPFLC YPITKKGINT ALSKLKSVVS GDILSYSIAG RNEVFSNKLI 2099
RSVA Nashville
RSVA Denver
RSVA Milwaukee
RSVA VR-26 Long
RSVA A2
RSVB Dallas
RSVB Milwaukee
RSVB 9320
RSVB strain B1
2,120
2,140
2,160
RSVA Memphis-37
NHKHMNILKW FNHVLNFRST ELNYNHLYMV ESTYPYLSEL LNSLTTNELK KLIKITGSLL YNFHNE* 2166
RSVA Nashville 2165
RSVA Denver 2165
RSVA Milwaukee 2165
RSVA VR-26 Long 2165
RSVA A2 2166
RSVB Dallas 2166
RSVB Milwaukee 2166
RSVB 9320 2166
RSVB strain B1 2166
